# Gene Expression Profile of Human Mesenchymal Stromal Cells Exposed to Hypoxic and Pseudohypoxic Preconditioning—An Analysis by RNA Sequencing

**DOI:** 10.3390/ijms22158160

**Published:** 2021-07-29

**Authors:** Katarzyna Zielniok, Anna Burdzinska, Victor Murcia Pienkowski, Agnieszka Koppolu, Malgorzata Rydzanicz, Radoslaw Zagozdzon, Leszek Paczek

**Affiliations:** 1 Department of Immunology, Transplantology and Internal Diseases, Medical University of Warsaw, Nowogrodzka 59, 02-006 Warsaw, Poland; radoslaw.zagozdzon@wum.edu.pl (R.Z.); leszek.paczek@wum.edu.pl (L.P.); 2 Department of Clinical Immunology, Medical University of Warsaw, Nowogrodzka 59, 02-006 Warsaw, Poland; 3 Department of Medical Genetics, Medical University of Warsaw, Pawińskiego 3C, 02-106 Warsaw, Poland; victor.murciapienkowski@wum.edu.pl (V.M.P.); agnieszka.jacoszek@wum.edu.pl (A.K.); malgorzata.rydzanicz@wum.edu.pl (M.R.); 4 Department of Bioinformatics, Institute of Biochemistry and Biophysics, Polish Academy of Sciences, Pawińskiego 5A, 02-106 Warsaw, Poland

**Keywords:** mesenchymal stem cells, MSCs, hypoxic priming, PHDs inhibitor, Vadadustat, HIF-1α, transcriptional regulation, RNA-Seq

## Abstract

Mesenchymal stromal cell (MSC) therapy is making its way into clinical practice, accompanied by research into strategies improving their therapeutic potential. Preconditioning MSCs with hypoxia-inducible factors-α (HIFα) stabilizers is an alternative to hypoxic priming, but there remains insufficient data evaluating its transcriptomic effect. Herein, we determined the gene expression profile of 6 human bone marrow-derived MSCs preconditioned for 6 h in 2% O_2_ (hypoxia) or with 40 μM Vadadustat, compared to control cells and each other. RNA-Sequencing was performed using the Illumina platform, quality control with FastQC and adapter-trimming with BBDUK2. Transcripts were mapped to the Homo_sapiens. GRCh37 genome and converted to relative expression using Salmon. Differentially expressed genes (DEGs) were generated using DESeq2 while functional enrichment was performed in GSEA and g:Profiler. Comparison of hypoxia versus control resulted in 250 DEGs, Vadadustat versus control 1071, and Vadadustat versus hypoxia 1770. The terms enriched in both phenotypes referred mainly to metabolism, in Vadadustat additionally to vesicular transport, chromatin modifications and interaction with extracellular matrix. Compared with hypoxia, Vadadustat upregulated autophagic, phospholipid metabolism, and TLR cascade genes, downregulated those of cytoskeleton and GG-NER pathway and regulated 74 secretory factor genes. Our results provide valuable insight into the transcriptomic effects of these two methods of MSCs preconditioning.

## 1. Introduction

Mesenchymal stromal cells (MSCs) are a promising candidate for widely used cell-based therapy. This therapy utilizes the body’s endogenous regenerative mechanism to repair function and restore physiological homeostasis in a broad spectrum of tissues affected by disease processes. While only ten MSCs-based therapeutics have currently been globally approved (listed in [1]), a vast number of MSCs therapies are under research and development for applications in a variety of diseases including but not limited to graft-versus-host disease (GvHD), several autoimmune diseases (rheumatoid arthritis, systematic lupus erythematosus, psoriasis vulgaris), neurodegenerative diseases (Alzheimer’s disease, multiple sclerosis, amyotrophic lateral sclerosis), cardiac disorders (acute myocardial infarction, ischemic stroke, chronic heart failure), kidney disorders (renal transplantation, diabetic nephropathy), inflammatory bowel diseases (Crohn’s disease) as well as COVID-19 (reviewed in [1]). MSCs belong to a population of adult progenitor cells that, in addition to their ability to self-renew and differentiate into cells with several mesenchymal phenotypes, are also characterized by their potential to be activated in response to tissue injury to promote regenerative processes, mainly through paracrine activity. In particular, MSCs secrete a number of biologically active compounds (cytokines, growth factors, antiapoptotic and antioxidative factors, extracellular matrix components, extracellular vesicles and even cellular organelles), constituting a major component coordinating regenerative processes associated with tissue healing [2]. MSCs are also distinguished by their ability to reduce excessive inflammatory processes and regulate both innate and adaptive immune responses. Reception of proinflammatory signals accumulating in the lesion area direct MSCs to secrete mediators that generally lead to suppression of CD4+ and CD8+ T cells, inhibition of NK and dendritic cell activation, while promoting the generation of regulatory T cells (Treg) and regulatory B cells (Breg) [3]. Due to the current state of their microenvironment, MSCs also dynamically regulate the expression of toll-like receptors (TLRs) 2, 3, 4, 7 and 9, so TLR signaling also affects their immune-related activity [3]. Moreover, MSCs exhibit a phenotype that promotes immune tolerance. They show very low expression of major histocompatibility complex Class I (MHC I) surface antigens and express MHC Class II only upon exposure to proinflammatory stimuli [4]. Additionally, MSCs possess reduced levels of major components of the antigen processing machinery (APM), lacking expression of costimulatory molecules such as CD80, CD86, CD40 or CD40L [3]. MSCs also gain immune tolerance through surface expression and secretion of PDL1 and PDL2 molecules [5], as well as secretion of sHLA-G (a non-classical MHC Class Ib antigen), which is known for its association with achieving fetal immune tolerance [6]. These processes involved in immune system regulation are now considered to have the major potential for MSCs therapy. However, despite promising in vitro studies demonstrating their potent immunomodulatory and tissue regenerative abilities, the benefits of MSCs administration to patients in clinical trials are less than expected [2]. Therefore, many studies are currently directed towards finding optimal culture methods or preconditioning agents to improve the function and survival of MSCs for clinical application. Many preconditioning strategies are associated with culturing MSCs in oxygen-reduced conditions that reflect those found in their natural niches. Furthermore, exposure to hypoxic or hypoxia-like conditions has been shown to improve migration, regenerative and proangiogenic potential as well as stemness of MSCs [7,8,9,10]. In our recent study, we demonstrated that inhibition of hypoxia-inducible factor prolyl hydroxylase 2 (PHD2) by Vadadustat (AKB-6548) regulated the immunomodulatory potential of human bone marrow-derived MSCs [11]. PHD2 is one of three isoforms of oxygen-dependent dioxygenases responsible for hydroxylation of two proline residues in Hypoxia-inducible transcription factors alpha (HIFα). HIF-PHDs, together with the action of factor inhibiting HIF (FIH), are responsible for regulating the activity of several transcription factors that adapt the cell to low oxygen supply under hypoxia. Thus, it is conceivable that pharmacological inhibition of only PHD2 may induce a different gene regulatory effect than that induced by physical hypoxia. While there are two papers comparing the transcriptome of cells exposed to hypoxia and stabilization of HIFs [12,13], they have been conducted using cell lines, mostly tumor ones. To the best of our knowledge, no such studies have been conducted on primary cells as MSCs. Therefore, in this study, to understand how preconditioning of BM-MSCs in hypoxia differs from preconditioning with Vadadustat, and to fully characterize the gene-regulatory effect caused by exposure of MSCs to these two preconditioning methods, we performed gene expression profiling using RNA sequencing (RNA-Seq). The presented analysis of gene expression profile of BM-MSCs allows the determination of how the use of two different hypoxic preconditioning methods affects the functionality of MSCs, thereby providing a better understanding of their physiological role and improving the safety of their use in clinical therapy.

## 2. Results

### 2.1. Isolation and Characterization of Human BM-MSCs

All six populations of BM-MSCs cells used in the study met the identification criteria according to the International Society for Cell and Gene Therapy (ISCT) statement established in 2006 [14]. They adhered to the plastic culture surface and formed colonies, showed expression of the surface markers CD73 (mean 99.5%), CD90 (mean 99.0%), and CD105 (mean 99.4%) and the absence of CD45, CD34, CD11b, CD19, and HLA-DR antigens in 98.3% of the cells, and demonstrated the ability to differentiate into cartilage, bone, and adipose tissue cells. Detailed results of identification analyses of the six BM-MSCs populations used in this study have been published previously in Zielniok et al. (2020) (Supplementary Tables S1–S3, donors 1–6 in [11]).

### 2.2. Transcriptional Signatures and Major Differences between the Response of MSCs to Two Hypoxic Preconditioning Methods

**Figure 1:**
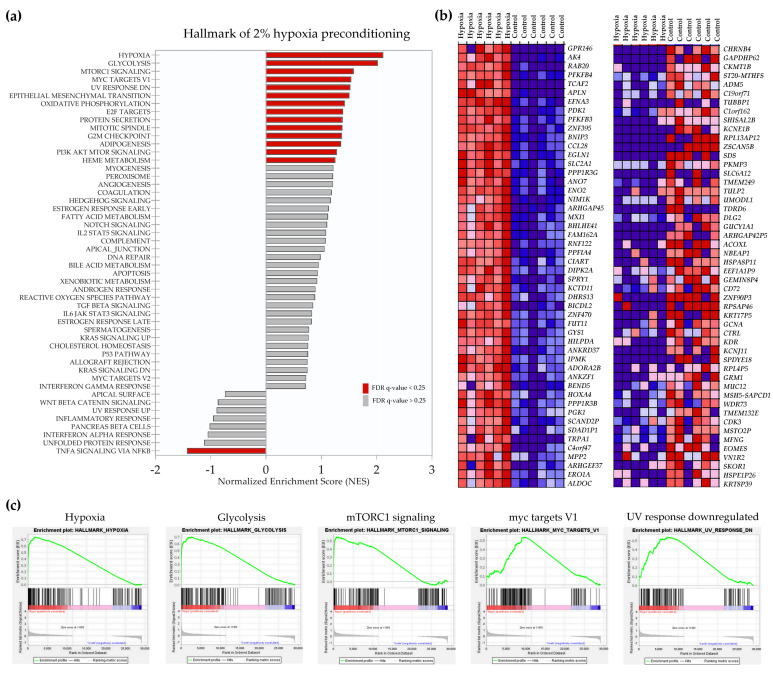

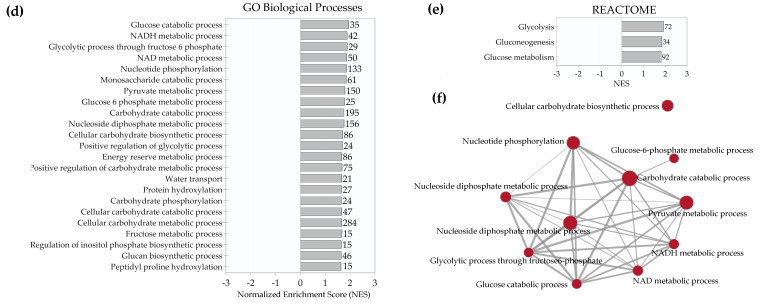
Gene set enrichment analysis (GSEA) results of 2% hypoxia vs. control comparison. RNA-Seq was performed on samples collected after 6 h preconditioning of six human BM-MSCs populations with 2% O_2_ (hypoxia) compared to cells cultured in 20% O_2_ (control). (**a**) Results of GSEA Hallmark analysis showing enriched gene sets. Bars in red indicate significant enrichment at FDR < 25%, bars in gray represent gene sets with FDR > 25% and a nominal *p* value < 5%. A positive Normalized Enrichment Score (NES) value indicates enrichment in the hypoxia phenotype, a negative NES indicates enrichment in the control phenotype. (**b**) Heat map of the top 50 marker genes for each phenotype in the comparison of 2% hypoxia (left column) vs. control (right column). Expression values are represented as colors and range from red (high expression), pink (moderate), light blue (low) to dark blue (lowest expression) (**c**) Enrichment plots for top five data sets enriched in GSEA Hallmark analysis, showing the profile of the running ES Score and positions of gene set members on the rank-ordered list (**d**) Gene sets significantly enriched (FDR *q*-val < 0.25) in the hypoxia phenotype using the Gene Ontology Biological Processes, ordered by NES with the number of genes assigned to each gene set reported next to the bar (**e**) Gene sets significantly enriched (FDR *q*-val < 0.25) in hypoxia phenotype using the REACTOME database, ordered by NES with a number of genes assigned to each gene sets shown next to the bar (**f**) Enrichment map for the GSEA GO BP analysis results created in Cytoscape.

RNA-Seq identified global changes in gene expression of human BM-MSCs preconditioned with hypoxia (2% O_2_) or with 40 μM Vadadustat when compared to control cells incubated in 21% O_2_. Gene set enrichment analysis (GSEA) of normalized counts of all genes expressed in 2% hypoxia compared with those expressed in control conditions using the Hallmark database showed that 13 gene sets were significantly enriched (FDR < 25%) in hypoxia and one gene set was significantly enriched in control (Figure 1a).

**Figure 2:**
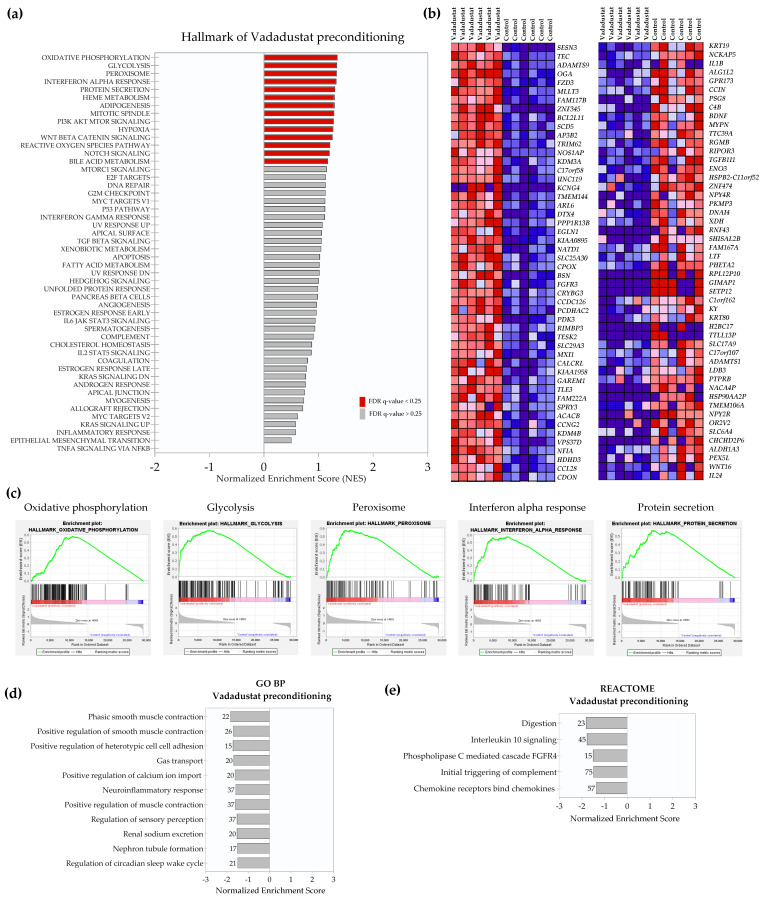
Gene set enrichment analysis (GSEA) results of Vadadustat vs. control comparison. RNA-Seq was performed on samples collected after 6 h preconditioning of six human BM-MSCs populations with 40 μM Vadadustat compared to cells cultured in 20% O_2_ (control) (**a**) Results of GSEA Hallmark analysis showing enriched gene sets. Bars in red indicate significant enrichment at FDR < 25%, bars in gray represent gene sets with FDR > 25% and a nominal *p* value < 5%. A positive normalized enrichment score (NES) value indicates enrichment in the Vadadustat phenotype. (**b**) Heat map of the top 50 marker genes for each phenotype in the comparison of Vadadustat (left column) vs. control (right column). Expression values are represented as colors and range from red (high expression), pink (moderate), light blue (low) to dark blue (lowest expression) (**c**) Enrichment plots for top five data sets enriched in GSEA Hallmark Vadadustat vs. control analysis, showing the profile of the running ES Score and positions of gene set members on the rank-ordered list (**d**) Gene sets significantly enriched (FDR *q*-val < 0.25) in the comparison of Vadadustat vs. control using the gene ontology biological processes, ordered by the increasing NES (a negative NES indicates enrichment in the control phenotype) with the number of genes assigned to each gene set given next to the bar (**e**) Gene sets significantly enriched (FDR *q*-val < 0.25) in the comparison of Vadadustat vs. control using the REACTOME database, ordered by NES (a negative NES indicates enrichment in the control phenotype) with a number of genes assigned to each gene sets shown next to the bar.

The five most highly enriched categories in hypoxia include “hypoxia”, “glycolysis”, “mTORc signaling”, “myc targets V1” and a category of genes downregulated in UV response (Figure 1c). In contrast, the “TNFA signaling via NFkB” is enriched under control conditions compared to hypoxia. Of the 29138 genes in the dataset, 11460 (39.3%) were identified as markers of hypoxia. A list of top 50 gene markers for the hypoxia versus control comparison is shown as a hierarchical heatmap in Figure 1b and includes *GPR146* (G Protein-Coupled Receptor 146), *AK4* (adenylate kinase 4), *RAB20* (RAB20, member RAS oncogene family), *PFKFB4* (6-phosphofructo-2-kinase/fructose-2,6-biphosphatase 4) and *TCAF2* (TRPM8 channel associated factor 2), as the first five genes most correlated with the hypoxia phenotype, and *CHRNB4* (cholinergic receptor nicotinic beta 4 subunit), *GAPDHP62* (glyceraldehyde 3 phosphate dehydrogenase pseudogene 62), *CKMT1B* (creatine kinase, mitochondrial 1B), *ST20-MTHFS* (ST20-MTHFS Readthrough) and *ADM5* (adrenomedullin 5 (putative)) as the first five marker genes correlated with control. Subsequently, the analysis using the gene ontology biological process (GO BP) database identified 22 processes positively enriched in the hypoxia phenotype (Figure 1d). Most of the processes were related to energy metabolism (starting with the most enriched “glucose catabolic process” and “glycolytic process through fructose-6-phosphate”), in addition to “protein hydroxylation” and “water transport”. When gene expression data acquired after MSCs preconditioning with 40 μM Vadadustat were similarly analyzed, different enriched categories than for hypoxia were obtained. GSEA analysis using the Hallmark database of gene expression data from Vadadustat preconditioning resulted in 14 gene sets significantly enriched at FDR < 25% (Figure 2a). 

**Figure 3:**
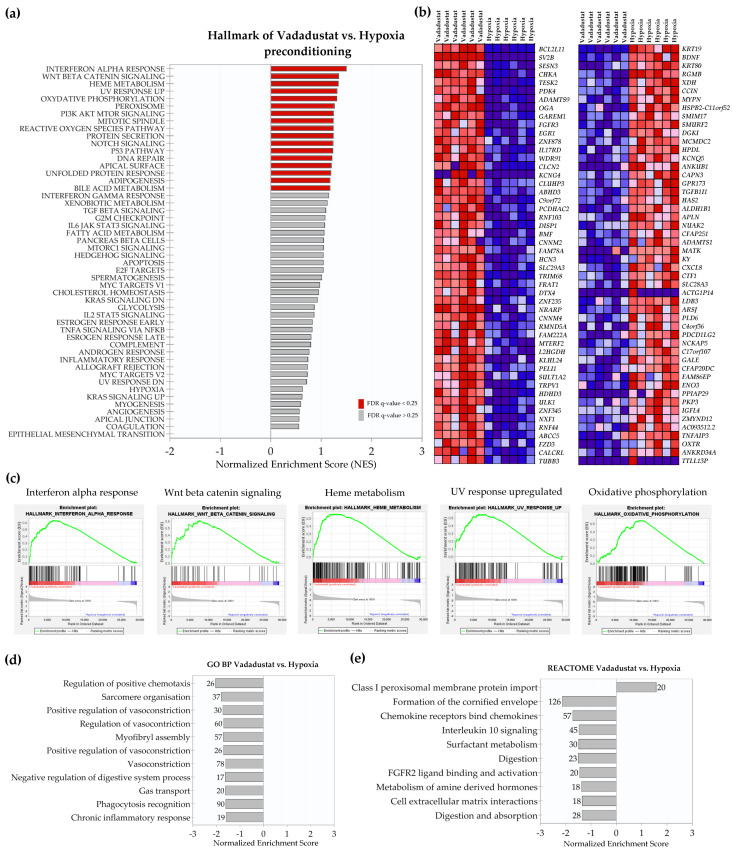
Gene set enrichment analysis (GSEA) results of Vadadustat vs. hypoxia comparison. RNA-Seq was performed on samples collected after 6 h preconditioning of six human BM-MSCs populations with 40 μM Vadadustat compared to cells preconditioned in 2% O_2_ (hypoxia) (**a**) Results of GSEA Hallmark analysis showing enriched gene sets. Bars in red indicate significant enrichment at FDR < 25%, bars in gray represent gene sets with FDR > 25% and a nominal *p* value < 5%. A positive normalized enrichment score (NES) value indicates enrichment in the Vadadustat phenotype. (**b**) Heat map of the top 50 marker genes for each phenotype in the comparison of Vadadustat (left column) vs. hypoxia (right column). Expression values are represented as colors and range from red (high expression), pink (moderate), light blue (low) to dark blue (lowest expression) (**c**) Enrichment plots for top five data sets enriched in GSEA Hallmark Vadadustat vs. hypoxia analysis, showing the profile of the running ES Score and positions of gene set members on the rank-ordered list (**d**) Gene sets significantly enriched (FDR q-val < 0.25) in the comparison of Vadadustat vs. hypoxia using the gene ontology biological processes, ordered by the increasing NES (a negative NES indicates enrichment in the control phenotype) with the number of genes assigned to each gene set given next to the bar (**e**) Gene sets significantly enriched (FDR q-val < 0.25) in the comparison of Vadadustat vs. hypoxia using the REACTOME database, ordered by NES (a positive NES indicates enrichment in Vadadustat phenotype, negative NES indicates enrichment in the hypoxia phenotype) with a number of genes assigned to each gene sets shown next to the bar.

The highest NES was obtained for the categories “oxidative phosphorylation” (1.35), “glycolysis” (1.33), “peroxisome” (1.33), “interferon alpha response” (1.32), and “protein secretion” (1.31) (Figure 2c). Of the 29138 genes in the dataset, 14,593 (50.1%) were assigned to the Vadadustat phenotype (with correlation area 84.7%). The top 50 marker genes for Vadadustat preconditioning are presented as a hierarchical heatmap (Figure 2b), among which the top five genes correlated with the Vadadustat phenotype were *SESN3* (sestrin 3), *TEC* (tec protein tyrosine kinase), *ADAMTS9* (ADAM metallopeptidase with thrombospondin type 1 motif 9), *OGA* (O-GlcNAcase), and *FZD3* (frizzled class receptor 3), and the top five genes correlated with control were *KRT19* (keratin 19), *NCKAP5* (NCK associated protein 5), *IL1B* (interleukin 1 beta), *ALG1L2* (ALG1 chitobiosyldiphosphodolichol beta-mannosyltransferase like 2) and *GPR173* (G protein-coupled receptor 173). Gene ontology enrichment analysis of biological pathways revealed no pathway positively enriched at FDR < 0.25 with the Vadadustat phenotype. Conversely, 11 gene sets were significantly enriched in the control phenotype, and mainly involved the categories of “smooth muscle contraction”, “cell-cell adhesion”, “gas transport” and “positive regulation of calcium ion import” (Figure 2d). Furthermore, analysis using the REACTOME database did not show the categories positively enriched in the Vadadustat phenotype either but identified five categories: “digestion”, “interleukin 10 signaling”, “phospholipase C mediated FGFR4 cascade”, “initial triggering of complement” and “chemokine receptors bind chemokines” as negatively correlated with Vadadustat phenotype (Figure 2e). In a final step, gene expression data of Vadadustat-preconditioned MSCs were compared directly to data from 2% hypoxia preconditioning. Comparative analysis of gene expression in MSCs preconditioned with Vadadustat vs. hypoxia using GSEA Hallmark indicated 17 gene sets significantly enriched in the Vadadustat phenotype at FDR < 25% (Figure 3a). The top five gene sets enriched in this analysis were the categories “Interferon alpha response” (NES = 1.52), “Wnt beta catenin signaling” (1.35), “Heme metabolism” (1.35), “UV response upregulated” (1.32), and “Oxidative phosphorylation” (1.31) (Figure 3c). Of the 29138 features in the dataset, 13931 (47.8%) genes were assigned to the Vadadustat phenotype (with correlation area 80.5%) and 15207 (52.2%) to the hypoxia phenotype (with correlation area 19.5%). Among the 50 marker genes for each phenotype shown in Figure 3b, the five highest correlated with the Vadadustat phenotype were *BCL2L11* (BCL2 like 11), *SV2B* (synaptic vesicle glycoprotein 2B), *SESN3* (sestrin 3), *CHKA* (choline kinase alpha), and *TESK2* (testis associated actin remodelling kinase 2). The top five most correlated with the hypoxia phenotype were *KRT19* (keratin 19), *BDNF* (brain-derived neurotrophic factor), *KRT80* (keratin 80), *RGMB* (repulsive guidance molecule BMP co-receptor b), and *XDH* (xanthine dehydrogenase). GSEA analysis using the GO BP database showed no gene set positively enriched with the Vadadustat phenotype, and 11 gene sets positively enriched with the hypoxia phenotype, of which “regulation of positive chemotaxis”, “sarcomere organization” and “positive regulation of vasoconstriction” had the highest NES, and the list was closed by the “chronic inflammatory response” gene set (Figure 3d). Analogous analysis using the REACTOME database identified one gene set enriched at FDR < 25% in the Vadadustat phenotype (“class I peroxisomal membrane protein import”), and nine gene sets enriched in the hypoxia phenotype (of which “formation of the cornified envelope”, “chemokine receptors bind chemokines”, and “interleukin 10 signalling” had the highest NES) (Figure 3e).

### 2.3. Preconditioning with Vadadustat Modulated BM-MSCs Gene Expression Profile to a Much Greater Extent Than Hypoxia

**Figure 4:**
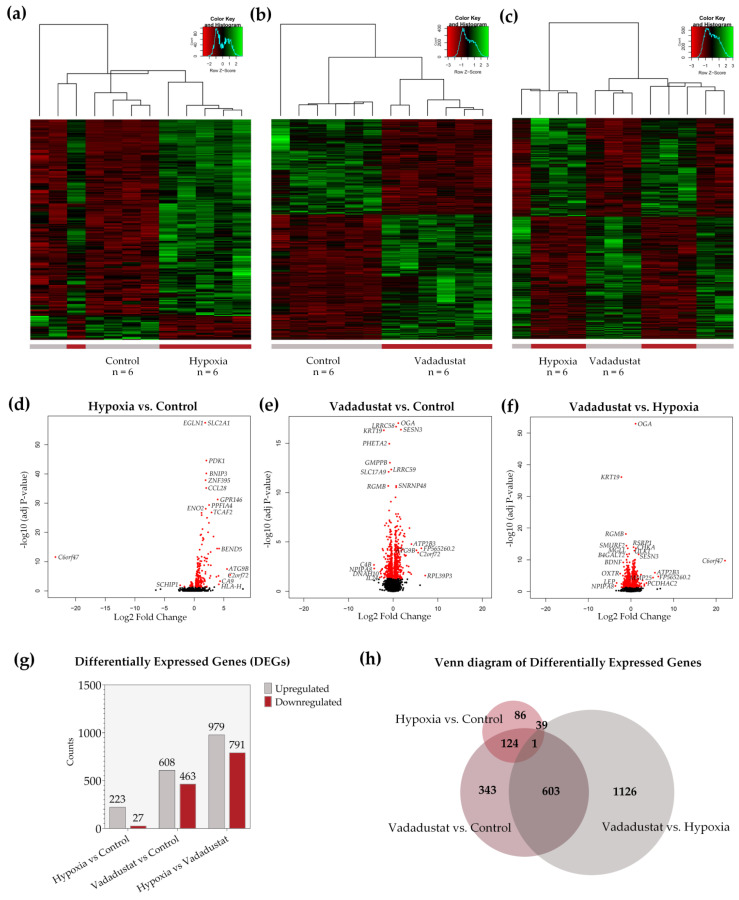
Analysis of differentially expressed genes (DEGs) in MSCs preconditioned with two hypoxic methods. Lists of DEGs were obtained by comparing the gene expression profile of six populations of hBM-MSCs preconditioned for 6 h in 2% O_2_ (hypoxia), preconditioned for 6 h with 40 μM Vadadustat, or cultured under 20% O_2_ (control). Hierarchical clustering heat map showing the correlation between DEGs (*p* < 0.05) for all pairwise combinations of samples in the dataset along with a hierarchical tree indicating the similarity between samples based on normalized gene expression values obtained from the comparison: (**a**) hypoxia vs. control (**b**) Vadadustat vs. control (**c**) Vadadustat vs. hypoxia. Volcano plot showing statistical significance of differential gene expression data (adjusted *p*-value) versus magnitude of expression change (log2 Fold Change) from comparison: (**d**) hypoxia vs. control, (**e**) Vadadustat vs. control, (**f**) Vadadustat vs. hypoxia; with significantly DEGs highlighted in red and the names of the most regulated genes highlighted next to the dots (**g**) The number of up- and downregulated DEGs obtained from all three comparisons (**h**) Venn diagram illustrating the number of unique and shared DEGs for each comparison.

In the next step, we analyzed the lists of differentially expressed genes obtained in DESeq2. Heatmaps and hierarchical clustering visualize transcriptomic differences between the three data comparisons: Hypoxia vs. control (Figure 4a), Vadadustat vs. control (Figure 4b) and Vadadustat vs. hypoxia (Figure 4c). 

Comparative gene expression analysis revealed dramatic differences in the number and profile of genes altered by preconditioning MSCs with the two hypoxia methods. Six-hour preconditioning of cells in 2% hypoxia resulted in significant changes in the expression of 250 genes compared to expression in control cells. Analogous treatment with Vadadustat resulted in 1071 DEGs compared to the gene expression of control MSCs and 1770 DEGs compared to gene expression of MSCs preconditioned in hypoxia (Figure 4g). Of the 250 genes differentially expressed in hypoxia, as many as 223 were upregulated and only 27 were downregulated. After Vadadustat preconditioning, this proportion was differently distributed; of the 1071 DEGs, 608 were upregulated and 463 were downregulated. Whereas the comparison of Vadadustat to hypoxia showed 979 DEGs upregulated and 791 DEGs downregulated. Analysis of lists of differentially expressed genes revealed that 603 DEGs were present in both the Vadadustat vs. control and Vadadustat vs. hypoxia comparisons (Venn diagram, Figure 4h). Exactly half of the genes significantly changed for the Hypoxia vs. control comparison (125 from 250 DEGs) were also changed in the Vadadustat vs. control comparison. 40 DEGs were common to the hypoxia vs. control and Vadadustat vs. hypoxia comparisons. Only one gene was common to all three comparisons. The distribution of all DEGs obtained for each treatment is shown in volcano plots (Figure 4d–f), mapped by Log2 Fold Change and adj *p*-value, with the gene names of the most significant and highest expression change listed. 

### 2.4. Enrichment Analysis of Biological Pathways Indicated Differences in the Effects of the Two Methods of Hypoxic Preconditioning of MSCs

**Figure 5:**
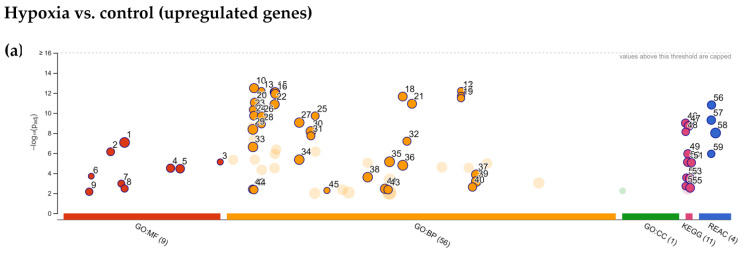

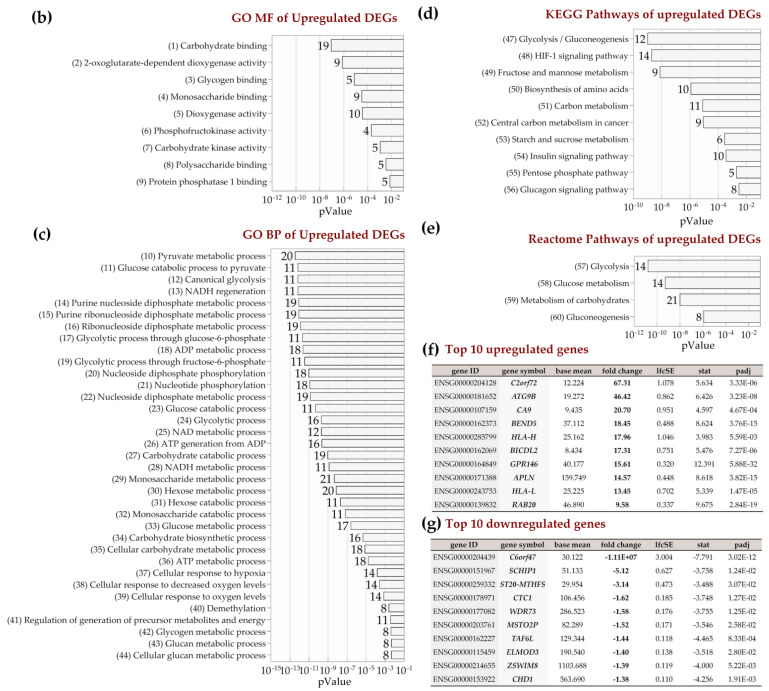
Functional enrichment analysis of the 223 upregulated differentially expressed genes (DEGs) between MSCs preconditioned in 2% O_2_ hypoxia and control cells using g:Profiler. The analysis was performed on samples obtained from six populations of hBM-MSCs preconditioned for 6 h in 2% O_2_ (hypoxia) compared to MSCs cultured under 20% O_2_ (control) (**a**) Results of enrichment analysis presented in the form of a Manhattan plot, where the *x*-axis shows the functional terms grouped by the color code of source database used, while the *y*-axis shows the enrichment adjusted *p*-values in negative decimal logarithm scale. Dots in the graph indicate all enriched terms meeting the significance criterion of *p* < 0.01, while highlighted dots represent terms filtered by the criterion of containing between 5 and 350 genes. The graphs (**b–e**) show the detailed results of the enriched terms highlighted in the Manhattan plot along with the statistical significance (pValue) and the number of DEGs belonging to the enriched term (placed next to the bar), according to the: (**b**) gene ontology molecular function (GO MF) (**c**) gene ontology biological processes (GO BP) (D) KEGG (E) REACTOME. (**f**) Table listing the top ten most highly upregulated genes, ranked by decreasing fold change in expression (bold values) including: Ensembl identifier (gene ID), gene symbol, the average of the normalized counts taken over all samples (baseMean), fold change in gene expression (fold change), standard error of the log2FoldChange estimate (lfcSE), Wald statistic (stat) and Benjamini–Hochberg adjusted *p*-value (padj). (**g**) Table listing the top 10 most highly downregulated genes, ranked by increasing fold change in expression (bold values) including: Ensembl identifier (gene ID), gene symbol, the average of the normalized counts taken over all samples (baseMean), fold change in gene expression (fold change), standard error of the log2FoldChange estimate (lfcSE), Wald statistic (stat) and Benjamini–Hochberg adjusted *p*-value (padj).

Analysis of lists of differentially expressed genes in terms of enriched gene ontology categories and biological pathways was conducted separately for upregulated and downregulated genes. The results obtained from the analysis of upregulated DEGs using the g:Profiler after preconditioning of MSCs with 2% hypoxia indicated major categories of gene expression enrichment according to the gene ontology molecular function (GO MF), biological process (GO BP), cellular compartment (GO CC) as well as KEGG and REACTOME databases (Figure 5a).

**Figure 6:**
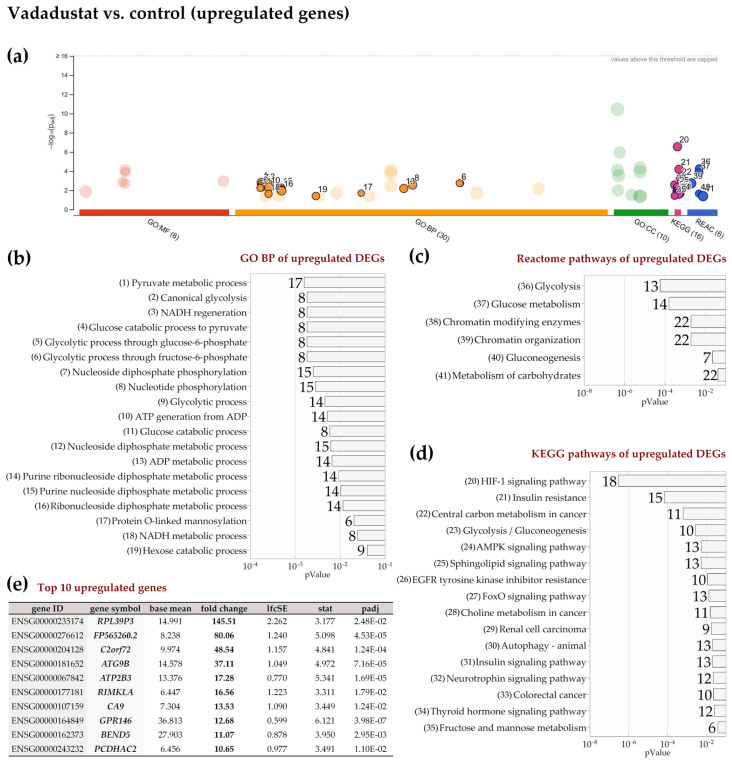
Functional enrichment analysis of 608 upregulated differentially expressed genes (DEGs) between MSCs preconditioned with Vadadustat and control cells using g:Profiler. The analysis was performed on samples obtained from six populations of hBM-MSCs preconditioned for 6 h with 40 μM Vadadustat compared to MSCs cultured under 20% O_2_ (control) (**a**) Results of enrichment analysis presented in the form of a Manhattan plot, where the *x*-axis shows the functional terms grouped by the color code of source database used, while the *y*-axis shows the enrichment adjusted *p*-values in negative decimal logarithm scale. Dots in the graph indicate all enriched terms meeting the significance criterion of *p* < 0.05, while highlighted dots represent terms filtered by the criterion of containing between 5 and 350 genes. The graphs (**b**–**d**) show the detailed results of the enriched terms highlighted in the Manhattan plot along with the statistical significance (pValue) and the number of DEGs belonging to the enriched term (placed next to the bar), according to the: (**b**) gene ontology biological processes (GO BP) (**c**) KEGG (**d**) REACTOME. (**e**) Table listing the top ten most highly upregulated genes, ranked by decreasing fold change in expression (bold values) including: Ensembl identifier (gene ID), gene symbol, the average of the normalized counts taken over all samples (baseMean), fold change in gene expression (fold change), standard error of the log2FoldChange estimate (lfcSE), Wald statistic (stat) and Benjamini–Hochberg adjusted *p*-value (padj).

Regarding the molecular functions of DEGs, the most significantly enriched categories were those related to carbohydrate binding, dioxygenase activity as well as phosphofructokinase activity (Figure 5b). Analysis of biological processes by GO BP revealed many enriched categories mainly related to energy metabolism: pyruvate metabolic process, canonical glycolysis, NADH/NAD metabolism, monosaccharide and glucan metabolism, but also nucleotide metabolism, cellular response to hypoxia and demethylation (Figure 5c). Glycolysis was also the most significantly enriched pathway according to the KEGG database (Figure 5d). Other categories of metabolism were also enriched by KEGG; in addition to fructose and mannose metabolism, central carbon metabolism in cancer, and amino acid biosynthesis, these included starch and sucrose metabolism and the pentose-phosphate pathway. Using the REACTOME pathways database, only metabolic categories were also indicated: glycolysis, glucose metabolism, gluconeogenesis, and pyruvate metabolism as shown in Figure 5e. A list of the top ten upregulated and downregulated genes after 2% hypoxia preconditioning are shown in Figure 5f,g, respectively). Due to the small number of genes significantly downregulated in hypoxia (only 27), no enrichment results were obtained from their analysis. When the list of upregulated DEGs after Vadadustat preconditioning was analyzed in g:Profiler, only in GO BP, REACTOME and KEGG databases significant enrichment was obtained (Figure 6a). 

**Figure 7:**
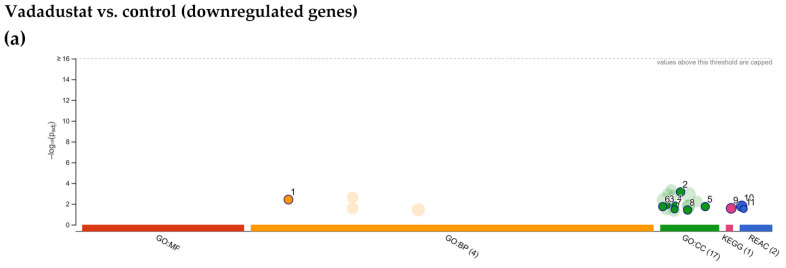

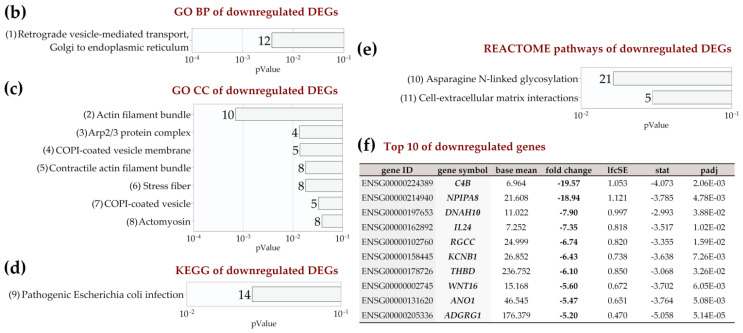
Functional enrichment analysis of 463 downregulated differentially expressed genes (DEGs) between MSCs preconditioned with Vadadustat and control cells using g:Profiler. The analysis was performed on samples obtained from six populations of hBM-MSCs preconditioned for 6 h with 40 μM Vadadustat compared to MSCs cultured under 20% O_2_ (control) (**a**) Results of enrichment analysis presented in the form of a Manhattan plot, where the *x*-axis shows the functional terms grouped by the color code of source database used, while the *y*-axis shows the enrichment adjusted *p*-values in negative decimal logarithm scale. Dots in the graph indicate all enriched terms meeting the significance criterion of *p*-values < 0.05, while highlighted dots represent terms filtered by the criterion of containing between 5 and 350 genes. The graphs (**b**–**d**) show the detailed results of the enriched terms highlighted in the Manhattan plot along with the statistical significance (pValue) and the number of DEGs belonging to the enriched term (placed next to the bar), according to the: (**b**) gene ontology biological processes (GO BP) (**c**) gene ontology cellular compartment (GO CC) (**d**) KEGG (**e**) REACTOME. (**f**) Table listing the top ten most highly downregulated genes, ranked by increasing fold change in expression (bold values) including: Ensembl identifier (gene ID), gene symbol, the average of the normalized counts taken over all samples (baseMean), fold change in gene expression (fold change), standard error of the log2FoldChange estimate (lfcSE), Wald statistic (stat) and Benjamini–Hochberg adjusted *p*-value (padj).

Among the biological pathways significantly enriched, metabolic categories were also predominant: NADH regeneration, glucose metabolism (canonical glycolysis, catabolism of glucose to pyruvate, glycolysis through glucose-6-phosphate, glycolysis through fructose-6-phosphate), pyruvate metabolism, nucleoside and nucleotide phosphorylation, ATP generation, purine rybonucleoside diphosphate and nucleoside disphosphate metabolism, but also O-linked protein mannosylation (Figure 6b). The largest variety of enriched categories was obtained using the KEGG database. In addition to the most significant category HIF-1 signaling pathway, the insulin resistance pathway, central carbon metabolism in cancer, glycolysis/gluconeogenesis, AMPK signaling pathway, sphingolipid signaling pathway and others shown in Figure 6c were also obtained. Among the enriched categories, autophagy (13 genes upregulated), neutrophin signaling pathway (12 genes) and thyroid hormone signaling pathway (12 genes) appeared for the first time. Further analysis in the REACTOME database revealed that in addition to the highest enriched metabolic categories (glycolysis, glucose metabolism, gluconeogenesis, and carbohydrate metabolism), categories related to chromatin remodelling were also significantly changed (chromatin-modifying enzymes and chromatin organization) as shown in Figure 6d. Figure 6e lists the top ten genes that were most highly expressed after Vadadustat preconditioning. When the list of downregulated DEGs after Vadadustat preconditioning was subjected to the same analysis, significantly fewer enriched categories were obtained (Figure 7a). In the absence of categories from the GO MF database, GO BP indicated only retrograde vesicle-mediated transport, Golgi to endoplasmic reticulum (Figure 7b). More categories were obtained in GO CC, where downregulated genes were assigned to cellular compartments: actin filament bundle, Arp2/3 protein complex, COPI-coated vesicle membrane, contracile actin filament bundle, stress fiber, COPI-coated vesicle and actomyosin (Figure 7c). The KEGG database indicated only an enriched pathogenic Escherichia coli infection category (Figure 7d).

**Figure 8:**
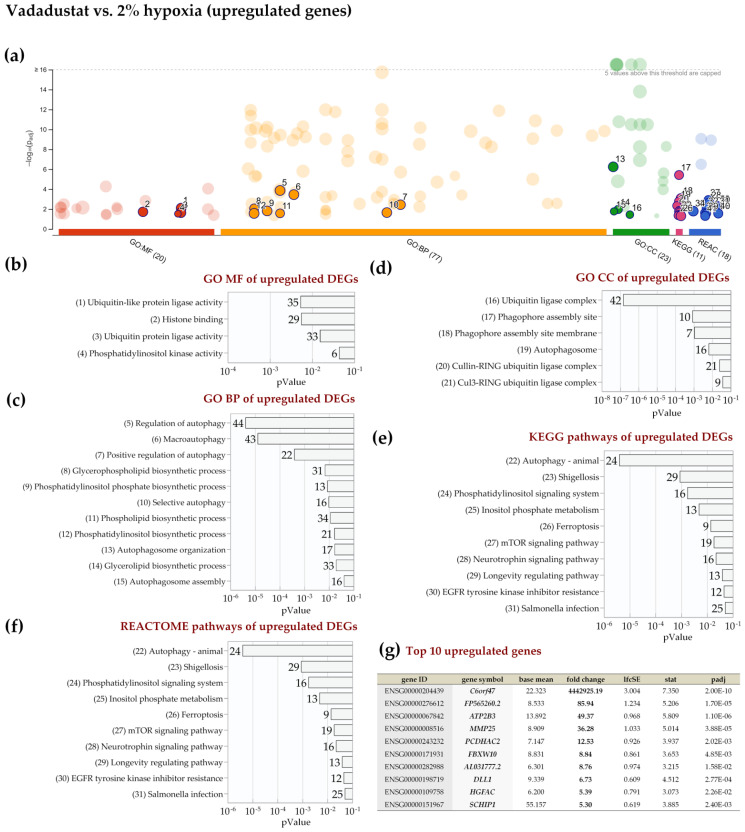
Functional enrichment analysis of 979 upregulated differentially expressed genes (DEGs) between MSCs preconditioned with Vadadustat and in 2% O_2_ hypoxia using g:Profiler. The analysis was performed on samples obtained from six populations of hBM-MSCs preconditioned for 6 h with 40 μM Vadadustat compared to MSCs cultured under 2% O_2_ (hypoxia) (**a**) Results of enrichment analysis presented in the form of a Manhattan plot, where the *x*-axis shows the functional terms grouped by the color code of source database used, while the *y*-axis shows the enrichment adjusted *p*-values in negative decimal logarithm scale. Dots in the graph indicate all enriched terms meeting the significance criterion of *p* < 0.05, while highlighted dots represent terms filtered by the criterion of containing between 5 and 350 genes. The graphs (**b**–**f**) show the detailed results of the enriched terms highlighted in the Manhattan plot along with the statistical significance (pValue) and the number of DEGs belonging to the enriched term (placed next to the bar), according to the: (**b**) gene ontology molecular function (GO MF) (**c**) gene ontology biological processes (GO BP) (**d**) gene ontology cellular compartment (GO CC) (**e**) KEGG (**f**) REACTOME. (**g**) Table listing the top ten most highly upregulated genes, ranked by decreasing fold change in expression (bold values) including: Ensembl identifier (gene ID), gene symbol, the average of the normalized counts taken over all samples (baseMean), fold change in expression (fold change), standard error of the log2FoldChange estimate (lfcSE), Wald statistic (stat) and Benjamini–Hochberg adjusted *p*-value (padj).

Based on the REACTOME database, it was determined that among the downregulated DEGs, a significantly larger number of genes than probability would result were assigned to the asparagine N-linked glycosylation and cell-extracellular matrix interactions categories (Figure 7d). Among the top ten downregulated genes after Vadadustat preconditioning (listed in Figure 7f), *C4B* (complement C4B), *NPIPA8* (Nuclear Pore Complex Interacting Protein Family Member A8), *DNAH10* (dynein axonemal heavy chain 10), and *IL24* (interleukin 24) were ranked most highly. Of particular interest, due to the comparison of the two types of hypoxic preconditioning, was the analysis of the list of DEGs between Vadadustat and 2% hypoxia, in which we obtained several enriched categories in each of the databases used in the g:Profiler analysis (Figure 8a). When analyzing the list of 979 upregulated differentially expressed genes, we found that the most significantly enriched categories by GO MF were ubiquitin (and ubiquitin-like) protein transferase and ligase activity and histone binding (Figure 8b). At the top of the list of enriched categories according to GO BP were autophagy terms (process utilizing autophagic mechanism, autophagy, regulation of autophagy, macroautophagy), and it should be noted that these categories were the most numerous in terms of genes from all enrichment analyses performed (Figure 8c). GO BP also indicated enrichments in the categories of glycerophospholipid metabolic process, positive regulation of cellular catabolic process, and phospatidylinositol phosphorylation. Regarding the Cellular Compartment, GO indicated categories for ubiquitin ligase complex, phagophore and autophagosome (Figure 8d).

**Figure 9:**
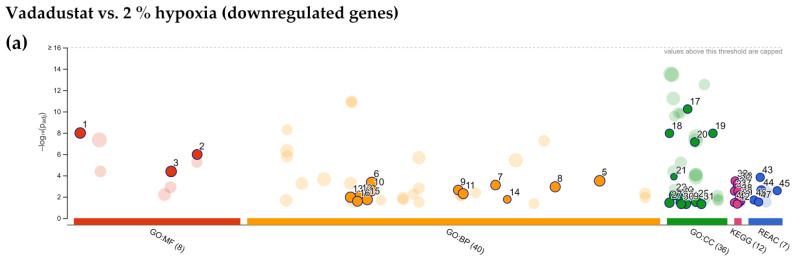

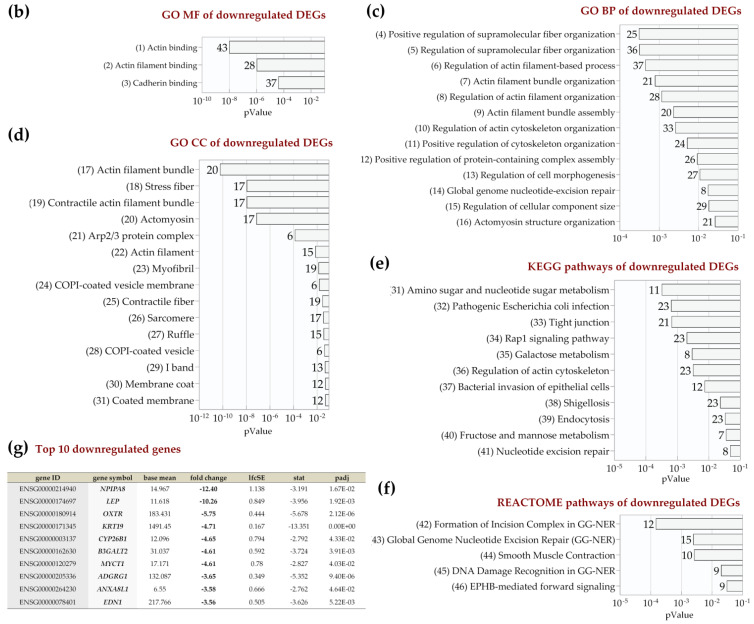
Functional enrichment analysis of 791 downregulated differentially expressed genes (DEGs) between MSCs preconditioned with Vadadustat and in 2% O_2_ hypoxia using g:Profiler. The analysis was performed on samples obtained from six populations of hBM-MSCs preconditioned for 6 h with 40 μM Vadadustat compared to MSCs cultured under 2% O_2_ (hypoxia) (**a**) Results of enrichment analysis presented in the form of a Manhattan plot, where the *x*-axis shows the functional terms grouped by the color code of source database used, while the *y*-axis shows the enrichment adjusted *p*-values in negative decimal logarithm scale. Dots in the graph indicate all enriched terms meeting the significance criterion of *p* < 0.05, while highlighted dots represent terms filtered by the criterion of containing between 5 and 350 genes. The graphs (**b**–**f**) show the detailed results of the enriched terms highlighted in the Manhattan plot along with the statistical significance (pValue) and the number of DEGs belonging to the enriched term (placed next to the bar), according to the: (**b**) gene ontology molecular function (GO MF) (**c**) gene ontology biological processes (GO BP) (**d**) gene ontology cellular compartment (GO CC) (**e**) KEGG (**f**) REACTOME. (**g**) Table listing the top ten most highly downregulated genes, ranked by increasing fold change in expression (bold values) including: Ensembl identifier (gene ID), gene symbol, the average of the normalized counts taken over all samples (baseMean), fold change in expression (fold change), standard error of the log2FoldChange estimate (lfcSE), Wald statistic (stat) and Benjamini–Hochberg adjusted *p*-value (padj).

Moreover, analysis based on the KEGG database also identified autophagy as the most significantly enriched pathway when comparing upregulated genes after preconditioning with Vadadustat to 2% hypoxia (Figure 8e). The enriched categories further include shigellosis, phosphatidylinositol signaling system, inositol phosphate metabolism, ferroptosis, and others, detailed in Figure 8e. Analysis using the REACTOME database, however, indicated that in addition to the highest significantly enriched categories of phosphatidylinositol (PI) and phospholipid metabolism, many pathways associated with toll-like receptors (TLRs) were enriched (Figure 8f). These include mainly the TLR3 cascade, and several categories related to the TLR4 signaling (in addition to the TLR4 cascade also TRIF-mediated TLR4 signaling and MyD88-independent TLR4 cascade). Furthermore, TLR5, TLR10, and TLR9 pathways were also listed. However, none of the genes encoding TLRs themselves were on the list of DEGs. REACTOME identified also categories related to chromatin organization and chromatin-modifying enzymes, MAP kinase activation, MyD88 cascade initiated on plasma membrane as well as HDACs deacetylate histones. At the top of the list of the ten most highly upregulated genes shown in Figure 8g were *C6orf47* (chromosome 6 open reading frame 47), *FP565260.2* (novel protein) and *ATP2B3* (ATPase plasma membrane Ca2+ transporting 3). The analysis of downregulated DEGs between Vadadustat and hypoxia preconditioning was also abundant in enriched categories, with results across all databases (Figure 9a). Regarding GO MF, categories related to actin/actin filament and cadherin binding were the most enriched (Figure 9b). Among the categories enriched by GO BP, terms related to regulation of cytoskeleton organization dominated (from positive regulation of supramolecular fiber organization, regulation of actin filament-based process, actin filament/cytoskeleton/filament bundle organization, positive regulation of cytoskeleton organization and actomyosin structure organization) (Figure 9c). Regulation of cell morphogenesis, global nucleotide-excision repair and regulation of cellular component size were also significantly enriched in GO BP analysis. The enrichment categories obtained in GO CC for downregulated DEGs were also mainly associated with cytoskeleton elements (actin/contracile actin filament bundle, stress fiber, actomyosin, Arp2/3 protein complex, myofibril, contracile fiber, sarcomere, ruffle, I band) and membranes (COPI-coated vesicle/vesicle membrane, coated membrane, membrane coat) (Figure 9d). The categories most enriched by KEGG between downregulated genes from the comparison of Vadadustat preconditioning to 2% hypoxia were amino sugar and nucleotide sugar metabolism, tight junction and pathogenic Escherichia coli infection (Figure 9e). Additionally, tight junction, Rap1 signaling pathway, galactose metabolism, regulation of actin cytoskeleton, two categories related to bacterial infection (bacterial invasion of epithelial cells and shigellosis), endocytosis and fructose and mannose metabolism were indicated. 

Based on REACTOME, only five categories among the list of downregulated DEGs were enriched, and these were: formation of incision complex in global genome nucleotide excision repair (GG-NER), GG-NER, smooth muscle contraction, DNA damage recognition in GG-NER and EPHB-mediated forward signaling (Figure 9f). Finally, the most downregulated genes in the list of DEGs between Vadadustat and hypoxia were *NPIPA8* (nuclear pore complex interacting protein family member A8), *LEP* (leptin) and *OXTR* (oxytocin receptor) (Figure 9g).

### 2.5. Vadadustat Preconditioning of BM-MSCs Affected the Expression of Genes Encoding Secreted Proteins

**Figure 10:**
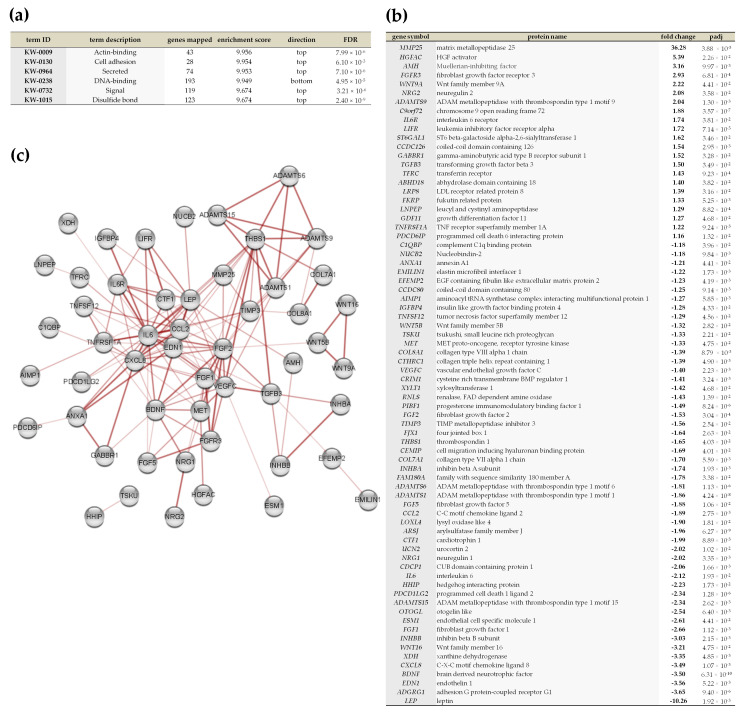
Analysis of differential expression of genes encoding secretory proteins between MSCs preconditioned with Vadadustat and 2% O_2_ hypoxia. (**a**) Results of annotated keywords (by UniProt) obtained using functional enrichment analysis in STRING 11.0 (FDR < 0.05), indicating enriched categories, number of genes mapped, enrichment score, gene location on input, and FDR value for enriched term. (**b**) Detailed list of 74 genes classified by Annotated keywords analysis into term “secreted” along with the names of the proteins they encode, fold changes in expression (bold), and the adjusted *p*-values. (**c**) Map of interactions between proteins encoded by genes classified to the term “secreted” in the annotated keywords analysis, created using protein-protein interaction analysis in STRING 11.0 (full network, score set to medium confidence and FDR < 0.05) visualized using Cytoscape software. Nodes represents proteins, while edges represents protein-protein association. The four edge thicknesses correspond to the confidence level from the thinnest at (0.15), through medium (0.4), high (0.7) to the thickest representing the highest (0.9) confidence, and its presence indicates i.e., that the proteins jointly participate in the shared function.

Analysis of DEGs by annotated keywords (Uniprot) in STRING did not reveal the term “secreted” (KW-0964) as enriched after MSCs preconditioning with 2% hypoxia. Conversely, analysis of DEGs after preconditioning with Vadadustat indicated enrichment for this term (Enrichment score 0.72, FDR 7.8 × 10^−4^), with 46 DEGs assigned (Supplementary Figure S1). The largest differences in the expression profile of genes encoding secretory factors were found when comparing preconditioning with Vadadustat to 2% hypoxia. The enriched term “secreted” (enrichment score 9.95, FDR 7.10 × 10^−6^, Figure 10a) included 74 genes (listed in Figure 10b), the vast majority of which remain functionally related (Figure 10c).

## 3. Discussion

Evaluation of genomic data is challenging. The key to understanding the vast amount of expression data is to assign features that allow them to be more easily interpreted. In our study, we used a standard approach to gene expression analysis that involves biological pathway profiling. This method, based on statistical tests for over-representation, identified those biological categories in which more genes were changed in expression as a result of MSCs preconditioning than would be expected by chance. Although the results of such an analysis cannot be directly translated into conclusions about the regulation of biological processes (since they involve regulation occurring at the level of genes, not proteins), this approach provides remarkable insight into the directions of changes initiated in cells in response to treatment. In the present study, we provided for the first time data on how expression of genes in BM-MSCs changes in response to short-term preconditioning with 2% hypoxia and pseudohypoxia (associated with pharmacological stabilization of HIF-1α by Vadadustat), and identified areas where these two responses differ. Previously, Elabd et al. (2018) presented an RNA-Seq analysis of gene expression changes in BM-MSCs cultured in 5% hypoxia compared to cells cultured in 20% O_2_ [15]. However, in this study, MSCs were cultured in hypoxia from the time of isolation for approximately 3 passages. Their analysis revealed that only 34 genes were differentially expressed between the transcriptome of MSCs cultured in hypoxia and in atmospheric O_2_, and none of these genes were directly related to energy metabolism. Our results identified as many as 250 differentially expressed genes between MSCs preconditioned with 2% hypoxia and cultured under control conditions (20% O_2_). The differences between the studies are most likely related to the oxygen tension used. While 5% O_2_ oscillates within the range of physiological bone marrow oxygen partial pressure (which is estimated to be 5.4–7% [16]), 2% more closely reflects hypoxic conditions. Moreover, our list of upregulated genes after hypoxia preconditioning of MSCs largely overlaps with the list of top 25 upregulated genes resulting from a meta-analysis of publicly available RNA-Seq data of 128 human and 52 murine hypoxia transcriptomes published by Bono and Hirota in 2019 [17]. 21 of the 25 genes presented by the authors were also identified in our analysis of DEGs under hypoxia. Our data showed that the exposure of MSCs to 2% hypoxic conditions predominantly activates the expression of genes related to energy metabolism (mainly glycolysis). And these findings are consistent regardless of the method and database we used in the analysis (GSEA vs. g:Profiler, GO BP vs. REACTOME). Furthermore, 6 h preconditioning of MSCs with Vadadustat compared to cells cultured under control conditions also induced changes mainly in the transcription of genes related to energy metabolism, which again included primarily glycolysis. The profile of changes in glycolytic gene expression was comparable between hypoxia and Vadadustat in both the type and magnitude of expression changes, as we reported previously in our commentary on the Roxadustat study [18]. However, it is important to emphasize that all these changes related to the expression of glycolysis and energy metabolism genes have a broader context than simply adapting the cell to a limited oxygen supply. In fact, MSCs in the undifferentiated state primarily exhibit a glycolytic phenotype [19], and glycolysis has been identified as the bioenergetic pathway that determines lifespan, genetic stability, migration, adhesion and proliferation of human MSCs [20,21]. Moreover, this upregulation of glycolytic genes is also relevant for MSCs immunomodulation and is primarily mediated by the transcription factor HIF-1α as demonstrated by Contreras-Lopez et al. (2020) [22]. The authors observed that knock-down for HIF-1α in murine MSCs (MSC_si_HIF1α) resulted in a metabolic switch from glycolysis to OXPHOS (reversed by oligomycin treatment), which in turn caused less effective inhibition of proliferation and differentiation of activated T-CD^4+^ cells into Th1 and Th17 lymphocytes. When the MSC_si_HIF1α were treated with oligomycin to restore glycolysis, their capacity of inhibiting proliferation of Th1 and Th17 was restored to a level of control MSCs. 

Many studies to date have demonstrated the extensive role that hypoxia and its associated transcriptional changes play in maintaining the therapeutic properties of MSCs [7,23,24,25,26]. And the fact that the transcription factor HIF-1α is a principal mediator of these changes is supported by positive results from studies involving its pharmacological stabilization [11,27,28,29]. However, in the present study, we showed that hypoxic preconditioning and pharmacological stabilization of HIF-1α by Vadadustat are markedly distinct in their effect at the gene expression level in MSCs. Analysis of the list of differentially expressed genes showed that these two methods of preconditioning differ more than the transcriptional effect of Vadadustat preconditioning does from the expression of control genes. From the comparative analysis of the results of Vadadustat vs. hypoxia preconditioning by GSEA, and which may affect the therapeutic potential of MSCs, we identified that while Vadadustat induces changes in the expression of genes belonging to different categories of signaling (interferon alpha response, Wnt beta catenin signaling, notch signaling), metabolism (oxidative phosphorylation, peroxisomal membrane protein import), and extracellular regulation (protein secretion), while categories related to immunomodulation (positive chemotaxis, IL-10 signaling, chemokine binding to chemokine receptor, chronic inflammatory response and cell extracellular matrix interaction) were enriched after hypoxia preconditioning. However, despite the enrichment results in the given categories, GSEA does not indicate the direction of changes in gene expression, containing both down- and upregulated genes. We obtained a slightly more detailed picture indicating the nature of the changes in expression by analyzing the list of differentially expressed genes (DEGs) between MSCs preconditioned with Vadadustat and hypoxia. This analysis indicated that Vadadustat preconditioning upregulates the expression of genes associated primarily with autophagy, as well as cellular catabolic processes, PI signaling, TLR signaling, chromatin organization, MAP kinase activation, and the longevity regulatory pathway. Conversely, it downregulated the expression of genes generally belonging to the two main categories: cytoskeleton organization and global genome nucleotide-excision repair. It is noteworthy that compared to both control MSCs and those in hypoxia, preconditioning with Vadadustat altered the expression of multiple genes encoding secretory proteins, including also several key factors for MSCs immunomodulation. Vadadustat caused changes in gene expression of secreted metalloproteinases: upregulation of *MMP25* and *ADAMTS9*, and downregulation of *ADAMTS1*, *ADAMTS6*, *MMP19*. Importantly, metalloproteinase activity (MMPs and ADAMs) is associated not only with cellular matrix remodeling but underlies normal immune processes, including facilitating leukocyte recruitment, participating in cytokine and chemokine processing (such as proteolytic cleavage of TNF, receptors for cytokines, adhesion molecules, Notch receptors, and many others) as well as defensin activation [30,31]. The metalloprotease MMP25, whose expression is very highly upregulated under Vadadustat preconditioning (both compared to control cells and especially to those preconditioned in hypoxia), may be involved in the regulation of the innate immune response by MSCs. Soria-Valles et al. (2016) have shown that MMP25 is engaged in activation of the NFκB pathway in leukocytes, being involved in the regulation of macrophage activation [32]. Furthermore, the authors demonstrated that Mmp25-null mice are less sensitive to LPS stimulation, exhibit hypergammaglobulinemia and reduced production of proinflammatory molecules. Moreover, among the Vadadustat-regulated genes encoding secretory proteins, upregulation of *HGFAC* (encoding hepatocyte growth factor activator) is also notable. HGFAC is a serine protease associated not only with HGF activation but also with macrophage stimulating protein (MSP) activation, playing an important role in macrophage recruitment to damaged tissues and their regeneration [33]. Finally, compared with hypoxia, Vadadustat caused downregulation of gene expression of key immunomodulatory factors such as *CCL2*, *IL6*, *PDCD1LG2*, *CXCL8*, and *LEP*, while upregulated the *TGFB3*, *IL6R* and *LIFR* genes. We had confirmed previously the regulation of these immunomodulatory factors by Vadadustat compared to control cells at the gene (using RT-PCR) and protein (using proteome profiler and Luminex) levels [11]. Therefore, the biological relevance of this regulation to the therapeutic activity of MSCs is discussed therein. The reason for such large differences in the gene expression profile between the two preconditioning methods is possibly due to the fact that hypoxia is associated not only with stabilization of HIF-1a through inhibition of hydroxylation of two proline residues (inhibition of PHDs activity), but also inhibition of hydroxylation of the asparagine residue by FIH (factor inhibiting HIF), as well as the activation of many other transcription factors (reviewed in [34]). 

Lastly, we would like to mention that the clinical use of PHDs inhibitors often raises concerns about their potential effects in promoting tumorigenesis, even though clinical trials generally do not confirm an increased incidence of cancer in treated patients (reviewed in [35]). While analysis of the list of differentially expressed genes between Vadadustat preconditioning and control cells using the KEGG database indicated upregulation of 9 genes associated with renal cell carcinoma and 10 genes associated with colorectal cancer, this was not surprising as it was already known that the HIF-1 pathway is upregulated in these two types of cancer [36,37]. Nevertheless, some concern may be raised by the results of the g:Profiler analysis between Vadadustat preconditioning and hypoxia, which indicated (using both the GO BP and REACTOME databases), that Vadadustat preconditioning caused downregulation of genes associated with global genome nucleotide excision repair (GG-NER). According to the REACTOME database, as many as 15 genes associated with this category and 9 genes in the DNA damage recognition in GG-NER were downregulated. These results may indicate an additional direction for investigating the distant adverse effects of treatment with PHD inhibitors. However, the regulation of these and all processes that were found to be enriched by our analyses of gene expression data, should be confirmed at the level of protein and biological processes activity. 

## 4. Materials and Methods

### 4.1. Isolation and Culture of Human Bone Marrow-Derived Mesenchymal Stromal Cells (BM-MSCs)

MSCs were isolated from bone marrow aspirates collected upon removal of the femoral head during hip endoprosthesis surgery of patients without a history of chronic disease. All samples were collected under the approval of the Bioethics Committee of Medical University of Warsaw (10 May 2016, No. KB/115/2016) after obtaining informed consent from each patient. Of the isolated MSCs that met the identification criteria, 6 populations from separate donors were randomly selected for the study. There were 5 populations isolated from males and 1 population isolated from a female. The mean age of the BM-MSCs donors was 50.5 years and the median was 44 years. All procedures were performed as we previously described [11,38]. The bone marrow samples were initially mechanically disassociated, then washed, centrifuged and suspended and seeded on a plastic culture dish (BD Primaria™, BD Biosciences, San Jose, CA, USA) in growth medium (low glucose DMEM, Biowest, Riverside, MO, USA) supplemented with 10% fetal bovine serum (Biowest, Riverside, MO, USA) and antibiotic-antimycotic solution (1% penicillin-streptomycin; 0.5% amphotericin B, invitrogen, Thermo Fisher Scientific, Waltham, MA, USA). After four days, when the first fibroblastic-like colonies of cells were observed growing at the bottom of the dish, the medium was changed and from then on it was changed routinely every second day. Routine MSCs cultures were maintained under conditions of 5% CO_2_, 95% humidified air at 37 °C. For the analyses, we used six BM-MSCs populations that were between passage four and six and that fulfilled acknowledged identification criteria for MSCs [14].

### 4.2. Methods for the Identification of Human BM-MSCs

The methods used to identify BM-MSCs were described in detail in our previous work [11]. We performed phenotyping of BM-MSCs on a flow cytometer (BD FACS Canto II using BD FACS Diva Software (BD Biosciences, San Jose, CA, USA)) using the BD Stemflow™ hMSC Analysis Kit (BD Biosciences). We then confirmed the ability of the isolated MSCs to undergo adipogenic (Oil Red O staining), osteogenic (Alizarin red staining and determination of alkaline phosphatase activity) and chondrogenic (Masson trichrome and toluidine blue staining of paraffin sections) differentiation.

### 4.3. Two Methods Used for Hypoxic Preconditioning of BM-MSCs

In the current study, two hypoxic preconditioning methods were used. The first, “physical” hypoxia was achieved by culturing cells for 6 h in a gas mixture composed of 2% O_2_ (balanced with N_2_) and 5% CO_2_. The second, “pharmacological” hypoxia was obtained by culturing cells for 6 h with the PHDs inhibitor, Vadadustat (AKB-6548, Akebia, Cambridge, MA, USA) in a concentration of 40 μM. The incubation time for gene expression analysis was chosen based on preliminary RT-PCR time-course analysis of two HIF-1α-related genes (*VEGF* and *GLUT1*) to determine the time point at which their expression increased most following exposure to experimental factors. The 2% O_2_ concentration was chosen to be lower than the physiological bone marrow oxygen partial pressure of 3–5% (reflecting hypoxia for BM-MSCs) but without causing anoxia. Vadadustat concentration was selected based on MTT assay [11] and Western blot analysis of HIF-1α level to achieve HIF-1α stabilization similar to that in 2% O_2_ hypoxia with minimal effect on cell metabolic activity. Vadadustat was prepared as a 5 mM stock solution in DMSO (Merck, Darmstadt, Germany) according to the manufacturer’s instructions, meaning that no more than 0.8 % (*v*/*v*) DMSO was present in the culture medium (without causing a noticeable cytotoxic effect in the MTT assay [11]). After 6 h of incubation, cells were harvested, washed with PBS (Merck, Darmstadt, Germany) and frozen at −80 °C for further analysis. The incubation time was experimentally chosen as optimal to visualize transcriptomic changes associated with hypoxic preconditioning of MSCs.

### 4.4. RNA Isolation from BM-MSCs

For RNA isolation, we cultured six populations of BM-MSCs between passages four and six on 60 mm dishes (BD Primaria™, BD Biosciences, San Jose, CA, USA) under standard growth conditions until a confluence of approximately 70% was reached. Then, incubation was started under experimental conditions: the first group was cultured in 2% O_2_ (physical hypoxia), the second group was supplemented with 40 μM Vadadustat in 21% O_2_ (pharmacological hypoxia), and the third was a control group growing under standard conditions (21% O_2_). The incubation lasted for 6 h, after which we removed the medium from the cell cultures, washed cells with PBS and disrupted them by scraping in 350 μL of RLT isolation buffer from Qiagen RNeasy Mini Kit (Qiagen, Hilden, Germany). The cell lysates were then frozen at −80 °C for further use. We extracted total cellular RNA from BM-MSCs using the RNeasy Mini Kit (Qiagen, Hilden, Germany) according to the protocol provided by the manufacturer. We determined the concentration and integrity of the isolated RNA samples using NanoDrop 1000 (NanoDrop Technologies, Thermo Fischer Scientific, Waltham, MA, USA) and Bioanalyzer Agilent RNA 6000 Nano kit (Agilent Technologies, Santa Clara, CA, USA).

### 4.5. Differential Gene Expression Analysis by RNA Sequencing (RNA-Seq) in BM-MSCs

We performed RNA-Seq on high-quality total RNA samples (RIN above 8.5) from BM-MSCs populations from six donors, isolated after 6 h of incubation: under standard growth conditions (control), in hypoxia (2% O_2_) or with 40 μM Vadadustat. cDNA libraries construction was carried out with 1 μg of total RNA using TruSeq Stranded mRNA Library Prep Kit (Illumina, San Diego, CA, USA) according to the manufacturer’s instruction, followed by paired-end sequencing (2 × 100 bp) using the HiSeq1500 platform (Illumina, San Diego, CA, USA). For each library, an average of 16–20 million read pairs were generated. Quality control checks of the sequencing raw data was conducted with FastQC (BaseSpace Labs, Illumina, San Diego, CA, USA), while adapter-trimming was performed with BBDUK2 [39]. The relative expression of transcripts were quantified for each donor using the Salmon v0.8.1 method [40]. Fastq files were mapped to the Homo_sapiens.GRCh37 reference genome using HISAT2 software version 2.1.0 [41]. The tximport pipeline [42] was used to import transcript abundance datasets for the differential gene expression analysis for three groups: control vs. Vadadustat, control vs. hypoxia and Vadadustat vs. hypoxia by DESeq2 software version 1.14.1 [43]. Hierarchical clustering was performed to display DEGs pattern using complete linkage and Euclidean distance as a measure of similarity, which, along with other RNA-Seq data visualizations, was performed using R packages (www.r-project.org).

### 4.6. Pathway Enrichment Analysis and Data Visualization

Pathway enrichment analysis was conducted according to the protocol of Reimand et al. (2019) [44]. To obtain a complete picture of gene expression changes associated with preconditioning of MSCs with physical and pharmacological hypoxia, we performed both ranked gene list analyses using Gene set enrichment analysis (GSEA) software version 4.1.0 [45] and DEGs list analysis using g:Profiler version e103_eg50_p15_eadf141 [46]. The first method analyzed transcript abundance from a ranked list of all available genes (whole-genome ranked list without using a cut-off) to identify regulation of functionally related gene sets with statistically significant enrichment. In the second method, the list of genes filtered by an FDR-adjusted *p* value threshold < 0.05 (with the exception for hypoxia, for which the adj *p* value < 0.01 was set due to extremely multiple results in GO analysis) was examined separately for up- and down-regulated genes. Based on the study of Hong et al. (2013) this approach is more powerful in identifying enriched categories corresponding to observed phenotypic differences in cells using pathway enrichment analysis [47]. Analyses in GSEA were performed on normalized counts generated in DESeq2. The analysis used h.all.v.7.4.symbols.gmt (Hallmark) and c5.all.v.7.4.symbols.gmt (Gene Ontology) gene set databases with the settings of perform; 1000 permutations, collapse dataset to gene symbols—false, permutation type—gene_set, enrichment statistic—weighted, metric for ranking genes—Signal2Noise, gene list sorting mode—real, gene list ordering mode—descending, max size of gene sets—500, and min size of gene sets—15. Functional enrichment of DEGs in g:Profiler was performed using the hsapiens (Human) genome version: GRCh38.p13, on the gene ontology molecular function (GO MF, annotations: BioMart, classes: releases/1 February 2021), gene ontology biological process (GO BP, annotations: BioMart, classes: releases/1 February 2021), gene ontology cellular compartment (GO CC, annotations: BioMart, classes: releases/1 February 2021), Kyoto encyclopedia of genes and genomes (KEGG, release 97.0 1 January 2021) and REACTOME (annotations: BioMart, classes: 2 December 2020) databases. Analysis was performed for annotated genes only. The g:SCS threshold was chosen as the significance threshold and the user threshold was set to *p* < 0.05. The analysis results were then filtered to display only terms sized between 5 and 350 genes as recommended in [44] and only these are highlighted and listed in the Manhattan plots generated by g:Profiler.

### 4.7. Analysis of Genes Encoding Secretory Proteins and Data Visualization

To demonstrate how the two used hypoxic preconditioning methods could potentially affect the secretory activity of MSCs, DEGs lists from hypoxia vs. control, Vadadustat vs. control, and Vadadustat vs. hypoxia comparisons were analyzed for Annotated Keywords (UniProt, FDR 0.05) using Functional Enrichment Analysis by STRING 11.0 [48]. Next, the list of genes assigned in the analysis to the “Secreted” category (KW-0964), was subjected to protein-protein interaction analysis in STRING software (full network, score set to medium confidence and FDR 0.05), and the resulting protein interaction map was uploaded to Cytoscape (version 3.8.2) for visualization [49].

## 5. Conclusions

Our study confirmed that exposure of MSCs to hypoxia initiates cellular adaptive processes through regulation of gene expression and that some of the regulated genes fit into common expression patterns among different human cells, but some are largely cell-specific. Furthermore, we have shown that activation of gene expression associated with hypoxia preconditioning can be partially achieved by preconditioning with pharmacological stabilizers of HIFα’s, but the two responses are substantially distinct. While hypoxia preconditioning caused moderate changes associated mainly with upregulation of genes related to metabolism (primarily energy), Vadadustat preconditioning resulted in much more extensive changes in regulated genes that were assigned to a variety of biological processes, from energy metabolism to chromatin modification, vesicular transport, asparagine glycosylation, or cell-extracellular matrix interactions. Comparative analysis of Vadadustat to hypoxia preconditioning indicated autophagy as the main term enriched with upregulated differentially expressed genes. This analysis also indicated upregulation of phospholipid metabolism, or TLR cascade genes, and signaling categories (MyD88 cascade or MAP kinase activation). Compared to hypoxia, Vadadustat downregulated genes in terms related to regulation of cell cytoskeleton and global genome nucleotide-excision repair. This study has provided a significant amount of data regarding the physiological response of MSCs to limited oxygen supply as well as the Vadadustat-mediated preconditioning method. Such large-scale data are important not only for indicating the direction of further research, but also contribute to a better understanding of the physiological function of MSCs, increasing the safety of their use in clinical therapy.

## Data Availability

RNA-Seq datasets generated during the current study are openly available in the NCBI Gene expresssion omnibus (GEO) repository, accession number GSE180371. Other datasets used and analyzed during the current study are available from the corresponding author on reasonable request.

## Supplementary Materials

The following are available online at https://www.mdpi.com/article/10.3390/ijms22158160/s1.
