# Supplementary figures and images for: Gene Expression Profile of Human Mesenchymal Stromal Cells Exposed to Hypoxic and Pseudohypoxic Preconditioning—An Analysis by RNA Sequencing
Source: Int J Mol Sci. 2021 Jul 29;22(15):8160. doi: 10.3390/ijms22158160 (PMC8348678; doi:10.3390/ijms22158160)

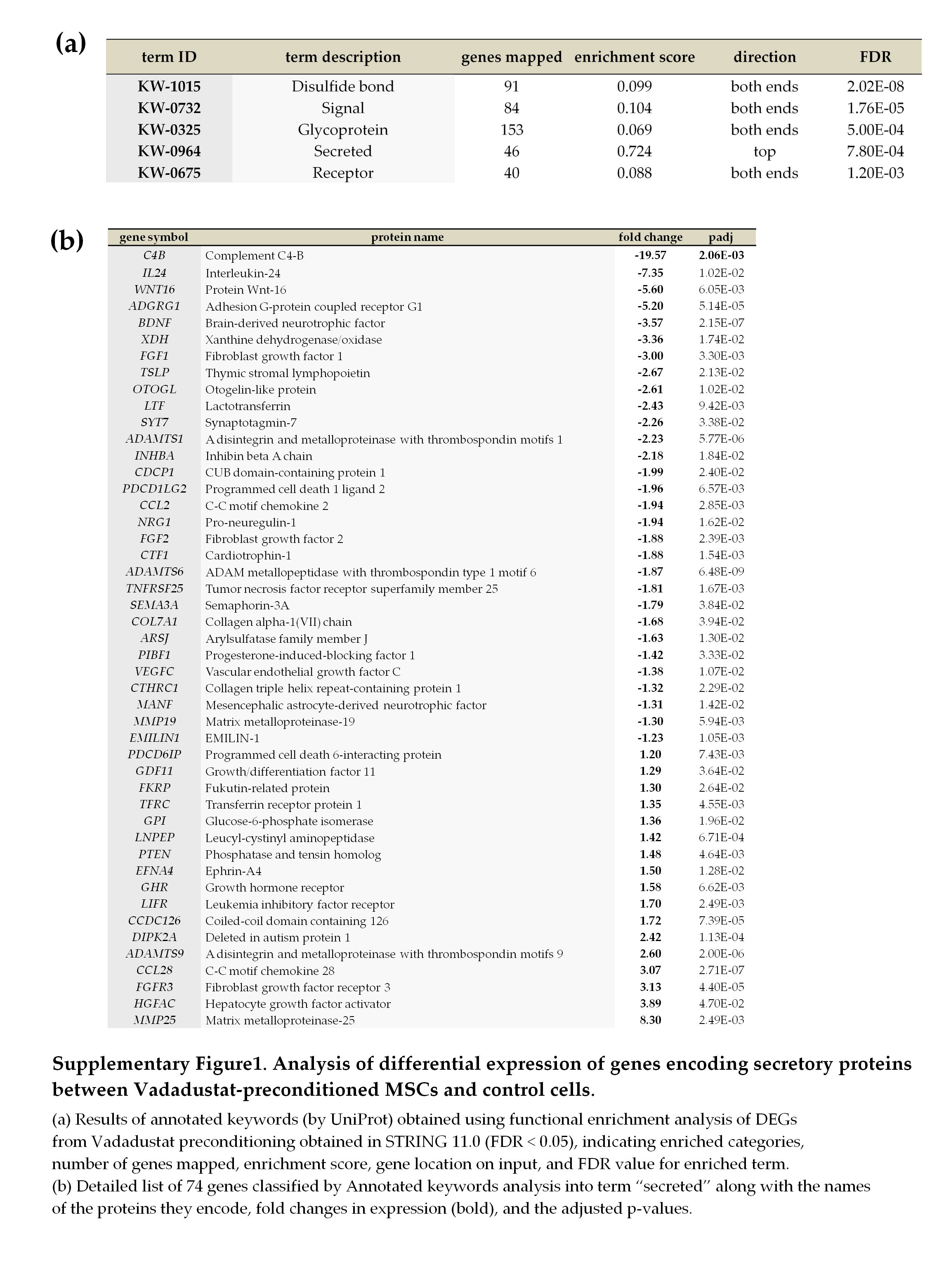

Supplement: Supplementary file 1 [file ijms-22-08160-s001.zip › Supplementary files/Supplementary Figure 1.tif]
